# A dual role for GRP in cardiovascular disease

**DOI:** 10.18632/aging.101851

**Published:** 2019-03-09

**Authors:** Carla S. B. Viegas, Dina C. Simes

**Affiliations:** 1 Centre of Marine Sciences (CCMAR), University of Algarve, Faro, Portugal; 2 GenoGla Diagnostics, Centre of Marine Sciences (CCMAR), University of Algarve, Faro, Portugal

**Keywords:** cardiovascular disease, Gla-rich protein (GRP), vascular calcification and inflammation, extracellular vesicles (EVs), calciprotein particles (CPPs)

Management and prevention of cardiovascular disease (CVD) represents one of the major health challenges worldwide. CVD is the leading cause of death globally despite all research efforts on last decades regarding the molecular mechanisms and processes involved on its development and progression. Chronic Kidney Disease (CKD) is an independent risk factor and promotor of CVD events, representing a considerable economic cost for the health system. CVD is the leading cause of death in all CKD stages, accounting for half the number of deaths in this population. In fact, cardiovascular risk is still incompletely explained by traditional risk factors, and risk factors such as increased chronic inflammation and vascular calcification (VC) have been identified as valuable prognostic tools for cardiovascular risk assessment [1]. Indeed, in the current view that highlights the complexity of CVD, inflammation and VC cannot be considered only hallmarks, but also drivers of disease progression functioning in a positive feed-back loop through a complex interplay with bidirectional crosstalk factors that influence CVD progression and outcomes [2]. A better understanding of this complex network and the discovery of new modulating agents targeting both inflammation and calcification, will pave the way to the discovery of new biomarkers and novel therapeutic strategies for cardiovascular-associated diseases. Gla-rich protein (GRP), a vitamin K-dependent protein, was shown to function as a calcification inhibitor in the cardiovascular system and as an anti-inflammatory agent in articular and immune cells [3–5], opening new perspectives for a crucial and global function as a modulator of calcification-related chronic inflammatory diseases such as CVD. Although GRP-deficient mice did not display a significant impairment in skeletal development, it was clearly demonstrated that vascular smooth muscle cells (VSMCs) from GRP^-/-^ mice, when exposed to calcifying conditions, have increased mineralization and expression of osteo/chondrogenic markers [6]. In addition to the inhibition of calcification at tissue level, we found that GRP is a constitutive component of circulating calciprotein particles (CPPs) and extracellular vesicles (EVs), and demonstrated that GRP is a systemic inhibitor of ectopic calcification through its involvement on the inhibition of Ca/P mineral crystal formation and maturation in blood, with profound consequences on VC [7]. In fact, although the role of mineral-containing EVs released from VSMCs has been recently highlighted as a nidus of calcification, the role and significance of circulating EVs in vascular calcification is still largely unknown. Also, the formation of CPPs has been suggested as a defense mechanism against Ca/P precipitation in blood, thereby capable of maintaining blood mineral homeostasis and prevent calcification [8]. Moreover, CPPs have attracted recent attention in clinical and basic research as a new molecular marker involved in CKD pathology. While several *in vitro* experiments have shown a deleterious effect of synthetic CPPs in cultured cells by promoting inflammatory and mineralization responses, we have shown that biological CPPs and EVs, isolated from CKD serum have an increased potential to induce calcification and inflammation in VSMCs. The capacity of these pathogenic circulating entities to promote VC is explained by a deficiency of GRP and fetuin-A, and a consequent increase in mineral maturation, when compared with nanoparticles from control healthy subjects [7]. Furthermore, removal of EVs from healthy serum clearly promoted VC, suggesting that these circulating nanoparticles containing higher levels of GRP and fetuin-A constitute a powerful anti-mineralization system that is able to regulate mineral formation both at systemic and tissue levels [7]. The relation between GRP levels in circulating EVs with CKD pathology and vascular calcification is certain of noteworthy importance from a translational perspective. EVs can be found in various tissues and fluids and considered to have an essential function in intercellular communication. They have also been regarded as attractive diagnostic biomarkers and their therapeutic use is an emerging field. While we previously demonstrated the presence of GRP in VSMCs and macrophage-released EVs, and its involvement in the mineralization-competence of VSMCs-derived EVs [3,4], we further shown that decreased levels of GRP in circulating EVs from CKD patients are correlated with increased pathogenicity of these nanostructures [7]. Although many questions are still opened and additional efforts are required to understand a possible relation between loading of circulating EVs and vascular health, from a biomarker perspective the lower levels of GRP in circulating EVs and CPPs foresee a potential application in CKD diagnostic and beyond.

Importantly, the dual capacity of GRP to act as an anti-inflammatory agent and an inhibitor of vascular calcification, was clearly demonstrated when the calcification/osteogenic differentiation and inflammatory status induced in VSMCs was rescued by supplementation of CPPs isolated from CKD patients with GRP [7]. This is in line with previous functional studies in human *in vitro* systems, showing that increasing GRP levels decrease extracellular matrix calcification and pro-inflammatory responses [3–5], strongly suggesting GRP as a potential therapeutic target for CVD.

Overall, we propose that GRP is a crucial component of a powerful anti-mineralization mechanism to regulate the dynamics of mineral formation both at systemic and tissue levels, to prevent pathological deposition of mineral and disease amplification through an increase in the inflammatory status. We are currently further exploring the role and mechanism of action of GRP as an anti-inflammatory agent on CVD. Since the interplay between calcification and inflammatory events are determinant processes to the development of CVD, possible approaches targeting the increase of GRP bioavailability might represent promising therapeutic options for vascular calcification-related diseases, and the determination of GRP levels in circulation should became a valuable tool for global cardiovascular risk assessment.
